# Text-book of Hygiene

**Published:** 1894-11

**Authors:** 


TEXT-BOOK OF HYGIENE. A Comprehensive Treatise on the Principles and Practice of Preventive Medicine from an American Standpoint. By George H. Rohé, M. D., Professor of Therapeutics, Hygiene, and Mental Diseases in the College of Physicians and Surgeons, Baltimore; Superintendent of the Maryland Hospital for the Insane; Member of the American Public Health Association; Foreign Associate of the Société Française d'Hygiène, etc. Third edition, thoroughly revised and largely rewritten, with many illustrations and valuable tables. Royal octavo, 553 pages. Cloth, $3.00 net. Philadelphia: The F. A. Davis Co., Publishers, 1914 and 1916 Cherry Street.

This book has been noticed in the pages of this journal before. A review of the first edition may be found in THE HOMŒOPATHIC PHYSICIAN for August, 1891, p. 340.

In that review it was commended except in one particular, and that was with regard to the feeding of cows on distillery "swill," against which a strong protest was recorded.

That protest has been gracefully ignored by the author, who, on pages 99 and 100, reiterates his disbelief in the injurious effect of "swill" from distilleries upon cows and their milk. It is incredible that any man with enough scientific knowledge to write such a book as this can, in the first instance, record his approval of this infernal practice of feeding cows on distillery and brewery refuse. Our protest in our previous review before referred to was emphasized by giving perfectly sound reasons for our objection.

The author says: "Much of the present agitation against 'swill milk' is more prompted by political demagogism than by scientific knowledge." Why, this "present agitation" has been going on for nearly forty years. The public were first made aware of the actual infamy of the swill milk business by *Frank Leslie's Illustrated Newspaper* nearly forty years ago. Artists visited the stables kept within the built-up parts of New York city and took sketches of the cows under every possible condition of health caused by the infamous food. Will our author say that the exposures made by that fearless newspaper were inspired by political demagogism? Certain it is that the descriptive articles published then, and the vivid illustrations accompanying them, have made an impression upon medical men that has never been forgotten, but which was the beginning of a salutary agitation which has almost effaced the shameful traffic from the whole population. The author of this book would seem to be starting an agitation which should bring about a revival of the evil.

A thing like this occurring in this otherwise estimable book, especially in this third edition, is calculated to throw distrust upon everything else the author says.

